# Effects of Vascular Endothelial Growth Factor 165 on Bone Tissue Engineering

**DOI:** 10.1371/journal.pone.0082945

**Published:** 2013-12-20

**Authors:** Lin Feng, Hao Wu, Lingling E, Dongsheng Wang, Fukui Feng, Yuwan Dong, Hongchen Liu, Lili Wang

**Affiliations:** 1 Oral Medical Research Center, Chinese PLA General Hospital, Beijing, P. R. China; 2 Department of Prosthodontics, Affiliated Stomatological Hospital of LMU, Jinzhou, P. R. China; University of Oxford, United Kingdom

## Abstract

To study the relationship between vascular endothelial growth factor (VEGF) and formation and repair of engineering bone, second-generation bone marrow stromal cells (BMSCs) of New Zealand white rabbits that were separated in vitro were transfected with VEGF 165 gene vectors by adenovirus to detect gene expressions. Transfected BMSCs and β-tricalcium phosphate material were complexed and implanted at the femoral injury sites of the study group (n = 12), and the control group (n = 12) were implanted with engineering bones that were not transfected with VEGF. Femoral recoveries of the two groups were observed on the 15th, 30th, 45th and 60th days, and their vascularization and ossification statuses were observed by immunohistochemical methods. The BMSCs transfected with VEGF highly expressed VEGF genes and excreted VEGF. The two groups both experienced increased vascularization and bone volume after implantation (t = 7.92, P<0.05), and the increases of the study group were significantly higher than those of the control group (t = 6.92, P<0.05). VEGF is clinically applicable because it can accelerate the formation and repair of engineering bone by promoting vascularization and ossification.

## Introduction

In clinical practice, bone grafting is devoted to repairing wide-range bone defects induced by trauma, tumor and infection. Therefore, it is crucial to further qualify and accelerate ossification to restore postoperative appearance and function. Currently, bone defects are commonly treated by autologous bone grafting, application of artificial substitutes and implantation of tissue engineering bones. Although the first three methods have long been used, they still suffer from disadvantages, thus allowing tissue engineering bone feasible in repairing large bone defects [1]–[3].

Tissue engineering bones usually lead to superb ossification and healing in repairing small bone defects, but they do not work well in case of large ones owing to necrosis in the center after ischemia. Meanwhile, natural bones survive and grow mainly depending on the sufficient bloods surrounding and inside that provide nutrients, active growth factors and oxygen, etc., and timely discharge metabolic wastes. Therefore, it is particularly important to establish a blood supply system before the formation of new bone tissues for bone defects, especially for the large ones [4], [5].

Although being spotlighted recently, implantation of tissue engineering bones may be prone to failure due to the death of BMSCs under hypoxic conditions [6]. Vascular endothelial growth factor (VEGF), which was first found and purified by Senger et al. in 1983 [7], is able to regulate the growth of blood vessels. Receptor-1 caters to recruiting hematopoietic stem cells, and receptor-2 is closely associated with vascularization. VEGF genes, which code VEGF, consist of 7 introns and 8 exons [8]. Being located at chromosome 6p21.3, VEGF genes are expressed as subtypes 121, 165 and 189 in human. As the dominant VEGF in human while being highly soluble, VEGF165 gene was transfected in BMSCs in this study to observe the ossification and vascularization results after complexation of transfected BMSCs and engineering bones, and to detail the relationship between VEGF and the formation and repair of engineering bones.

## Materials and Methods

This study was carried out in strict accordance with the recommendations in the Guide for the Care and Use of Laboratory Animals of the National Institutes of Health. The protocol was approved by the Committee on the Ethics of Animal Experiments of Chinese PLA General Hospital (Permit Number: 2012020548). All surgery was performed under anesthesia, and all efforts were made to minimize suffering.

### Experimental animals

Twenty-four New Zealand white rabbits were provided by the experimental animal center of Chinese PLA General Hospital, comprising 10 females and 14 males. They were aged 4–7 months old with the average age of 5.8. Their weights were 2.3–2.7 kg, with the average of 2.5 kg. They were then randomly divided into two groups (n = 12). Rabbits in the study group were implanted with the complex of BMSCs transfected with VEGF genes and tissue engineering bones, and those in the control group were implanted with blank engineering bones.

### Experimental design

**Figure 1:**
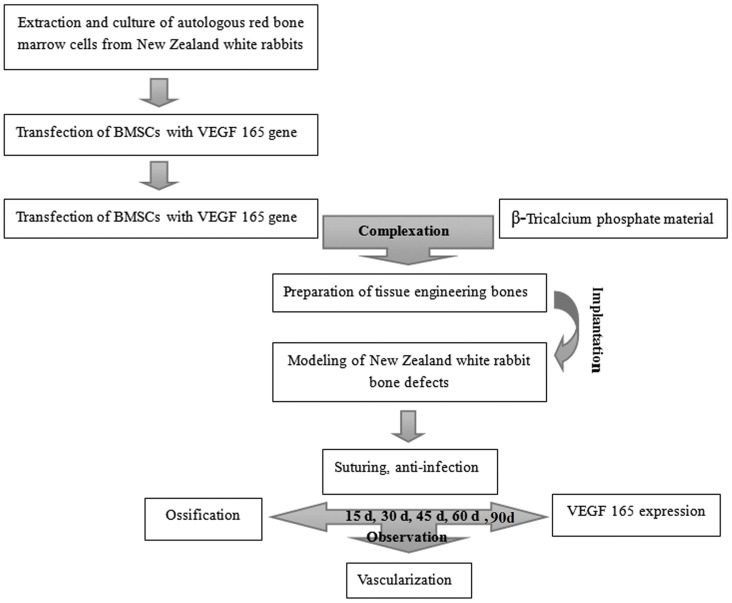
Design of experimental procedure.

The experiment procedure is schematized in Figure 1.

### Preparation of materials

Engineering bone (Shanghai Bio-lu Biomaterials Co., Ltd.): β-Tricalcium phosphate material, approximately 15 mm in length, cylindrical porous scaffolds, and 6 mm in cross-sectional radius; Fixation: Steel plate with screws; Reagents: DMEM culture medium, Trizol reagent, reagents for the extraction and amplification of genes such as adenovirus control vector, reagents for the transfection of cationic polymers, mouse anti-human VEGF 165 monoclonal antibody (primary antibody), and biotinylated goat anti-mouse antibody (secondary antibody) (Shanghai Touching Technology Co., Ltd.), etc.; Drugs: Anti-infective drugs such as sodium penicillin, anesthetics such as Sumianxin, ketamine and Atropine, and anticoagulants such as heparin; Others: PCR analyzer, absorbable threads, 5–0 silk threads, fretsaw, and syringe, etc.

### Experimental procedure

Recombinant shuttle plasmid pAdTrack/hVEGFl65 was established by PCR [9], and VEGF 165-adenovirus vector was then built, massively amplified and purified. Autologous red bone marrow cells of the rabbits from both iliac sides were cultured at 37°C and in 5% CO_2_ and passaged to the second generation until 70% of the cells fused. Then the cells were transfected with MOI = 150 for 12 h [10], cultured until 48 h and fixed in 95% ethanol. After inactivation of endogenous peroxidase by 0.3% H_2_O_2_, the cells were blocked with 10% goat serum, and the supernatant was discarded after 30 min. Expressions of VEGF 165 gene were detected by immunohistochemical methods with primary and secondary antibodies.

### Histological and morphometric analysis of in vivo studies

After 8 and 12 weeks, the harvested constructs were fixed in 10% formalin neutral buffer solution at pH 7.4 for 2 days and then decalcified with decalcifying solution composed of 10% hydrochloric acid (HCl) and 0.1% ethylene diamine tetraacetic acid (EDTA) for another 4 h. The specimens were then dehydrated through a series of graded ethanol, followed by infiltration and embedding into paraffin wax. The tissues were cut into 5 mm sections and observed using Toluidine blue staining. The same staining was performed in the con-trol. All the samples were analyzed microscopically and compared with controls. Digital photographs were recorded. For morphometric analysis, five sequential sections per implant were selected for evaluation under low magnification, allowing coverage of the entire implant. Using a Leica-Qwin 3.2 image analysis system (Leitz DMRD; Leica Microsystems Inc.), all sections were analyzed by an independent observer to identify the type of tissue (bone like or osteoid like). The extent of newly formed bone was indicated by the percentage of the newly formed bone areas within the section, and an average value was calculated for each implant. Data were then averaged across all implants within each group. Total scores per section were calculated and averaged for all sections to obtain an overall score for each implant. Data were then averaged across all implants within each group.

### Real-time quantitative PCR and RT-PCR

To investigate the underlying cellular and molecular mechanisms involved in the effect of VEGF165, real-time PCR and RT-PCR were used to quantify the levels of transcript-encoding factors in the two groups. After 14 days of culture, ALP, type I collagen and OCN mRNAs levels in the study group were up-regulated significantly. To isolate total RNA for reverse transcription-polymerase chain reaction (RT-PCR), the BMSCs were seeded on b-TCP at 1×10^5^cells/cm^2^ graft for 24 h to allow attachment in ordinary media. The cells were starved using a serum-free medium containing 2% bovine serum albumin, 100 U/Ml penicillin, and 100 mg/L streptomycin for 24 h. The cells were then cultured in media with/without 100 ng/mL VEGF165. After 14 days of culture, the BMSCs +β-TCP with/ without VEGF165 were crushed in lysis buffer (Roche) with an RNase-free piston (Pellet), shaken, and spun. The clear cell lysate was transferred to QiaShredder (Qiagen, Inc.) columns for RNA purification. RNase-Free DNase (Roche) was used to eliminate DNA contamination of RNA samples. Purified RNA was dissolved in RNase-free water and its concentration was assessed by reading at 260 nm. RNA quality was checked on a 2% agarose gel with 1 mg/mL ethidium bromide. Samples were stored at_808 C until use. Complementary DNA (cDNA) was synthesized from 2 mg of total RNA with the first-strand cDNA synthesis Kit for RT-PCR (AMV; Roche). Eight microliters of cDNA mixture diluted 1∶20 in water was subjected to real-time PCR using SYBR Green I dye (Lightcycler faststart DNA master SYBR green I; Roche). Reactions were performed in 20 mL PCR mixture containing 4 μL 5× Master Mix (dNTP mixture with dUTP instead of dTTP, MgCl_2_, SYBR Green I dye, Taq DNA Polymerase, and reaction buffer) and 2 mLof10 mM primers. Primer sequences (Shanghai Sangon Biological Engineering Technology and Services Co., Ltd.) of ALP, type I collagen, OCN, and β-actin are listed in Table 1. β-Actin real-time PCR was run as a control to monitor RNA integrity and to be used for normalization. Specificity of each primer pair was confirmed by melting curve analysis.

**Table 1:** Primers used in real-time polymerase chain reaction quantification and reverse transcription-polymerase chain reaction.

Gene	Primer sequence	Product size (bp)
ALP	F:5′-TCC CAC GTT TTC ACG TTT-3′	140
	R:5′-GAG ACG TTC CCT CGT TCA C -3′	
OC	F:5′-TGA GGA CCC TCT CTC TGC TC-3′	150
	R:5′-AGG TAG CGC CGG AGT CTA TT-3′	
COI	F:5′-CTA CAG CAC GCT TGT GGA TG-3′	192
	R:5′-ATT GGG ATG GAG CGA GTT TA-3′	
β-Actin	F:5′-CCC ATC TAT GAG GGT TAC GC-3′	152
	R:5′-TTT AAT GTC ACG CAC GAT TTC-3′	

### Vascular casting method

**Figure 2:**
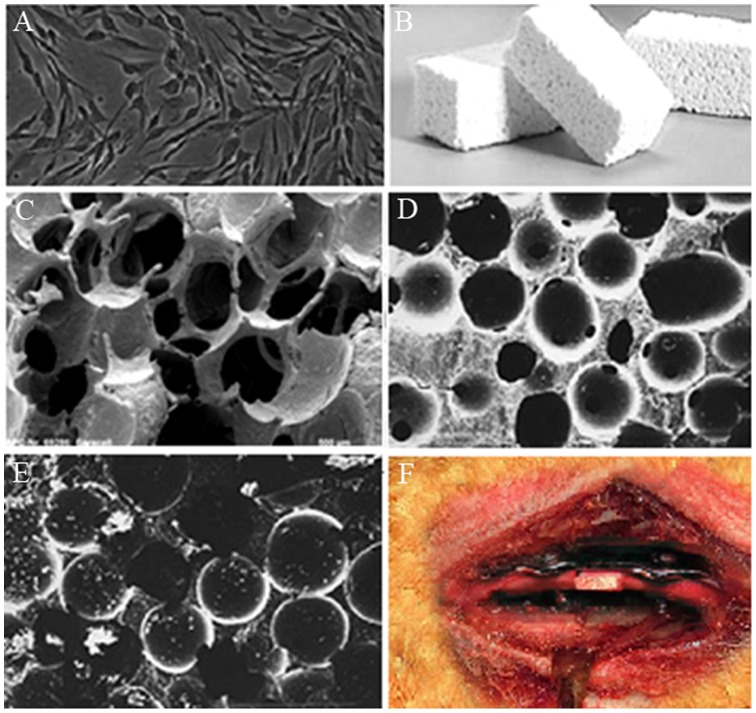
Vascular casting method. (A) BMSCs, ×200; (B) tricalcium phosphate material; (C) tricalcium phosphate under electron microscope, ×100; (D) tricalcium phosphate under electron microscope, ×40; (E) tricalcium phosphate-complexed BMSCs under electron microscope, ×100; (F) postoperative healing.

After being intravenously anesthetized with 0.03 ml/kg 1% sodium pentobarbital, the rabbits were incised on skins and subcutaneous tissues to find femoral arteries and veins that were centrifugally intubated and fixed. Benzoyl peroxide phthalocyanine (5 g), dimethylaniline (3 ml), dibutyl phthalate (35 ml) and appropriate amounts of red oil paint were then added in 300 ml of pre-polymerized methyl methacrylate. Once the polymerization began, blood vessels were infused until casting agents outflowed from veins that were then ligated. The infusion was continued until peripheral skins were completely stained red (250 ml of casting agents were used on each side) when infusion was terminated and arteries were ligated. As soon as the infused methyl methacrylate monomers underwent complete polymerization after blood vessels were cryogenically stored for 24–48 h while maintaining intravascular pressure, the samples were preserved and fixed in 10% formaldehyde solution for 2–3 weeks (Figure 2).

### Modified method of rabbit femoral defects [11]–[13]

Rabbits were anesthetized with Sumianxin, ketamine and Atropine, incised from anterolateral left hindlimb to expose the femur that was fixed by a 6-hole steel plate and reinforced by screws. A rabbit bone defect model was established by sawing an approximately 15 cm femur (the same as the length of β-tricalcium phosphate material), which was thereafter complexed with tissue engineering bones.

Periosteum and skin were successively sutured with absorbable threads and 5–0 silk threads, and the rabbits were injected with anti-infective drugs for 4 consecutive days (400000 U/d).

### Evaluation criteria

Positive expression of VEGF 165 gene [14]: By using the streptavidin-biotin complex method, positive expression rate was calculated based on the cells stained brownish red.

Ossification: Osteogenesis statuses at the femoral defects of the two groups were observed by X-ray on the 30th, 45th and 60th days. Parts of the specimens were subjected to HE [15] and Masson [16] staining, and the ossification results were observed after fixation and embedding.

Vascularization [17]: Neovascular endothelial cells at the bone defects of remaining specimens were marked by anti-rabbit CD31 antibody. The sections were observed under a high-magnification microscope, and 5 clear fields were selected to calculate the number of microvessels.

### Statistical analysis

All data were analyzed by SPSS 17.0. Two groups were compared by t test. P<0.05 was considered statistically significant.

## Results

### VEGF 165 gene expression after transfection

**Figure 3:**
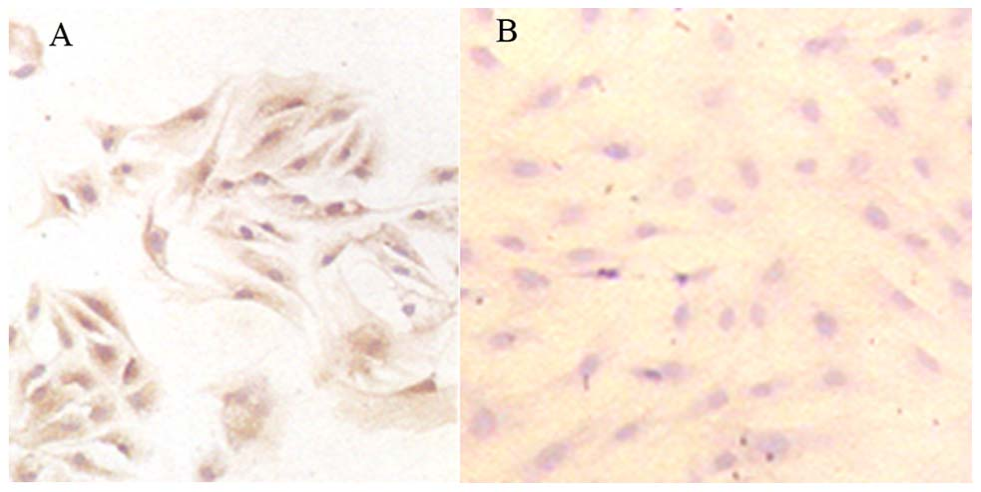
VEGF 165 gene expression after transfection. (A) Positive expression of VEGF in study group (10×10); (B) negative expression of VEGF in control group (10×10).

Under high-magnification microscope (10×10), the cells expressing VEGF were stained brown (Figure 3A), and the average positive expression rate of VEGF 165 gene was 92.14%. In other words, BMSCs that were transfected with adenovirus-mediated VEGF highly expressed VEGF. In contrast, the cells that did not express VEGF were not stained (Figure 3B).

### Ossification results

**Figure 4:**
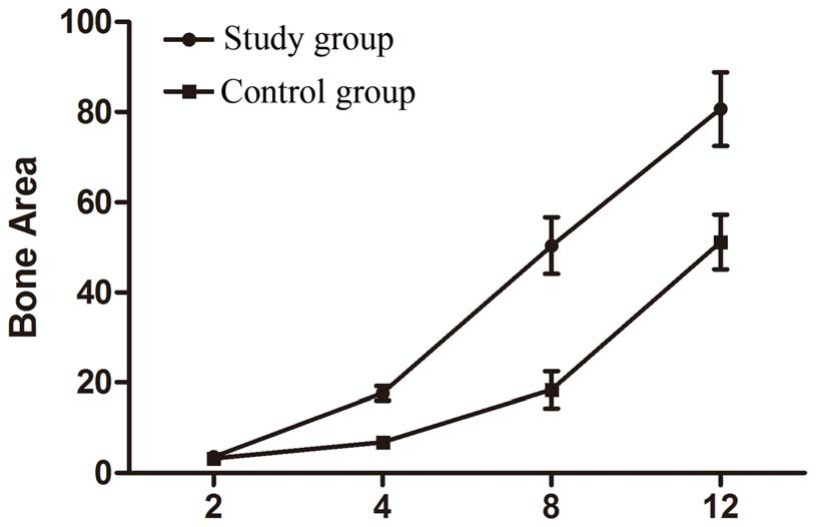
Relative area ratios of new bones (%).

**Figure 5:**
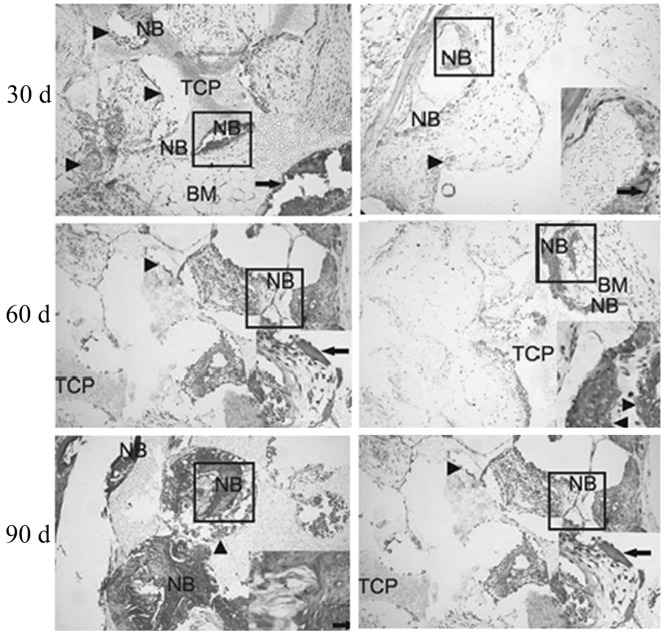
Ossification results on the 30th, 60th and 90th days (×100). Left: study group; right: control group. NB: new bone; BM: bone marrow; Arrowheads indicate blood vessels, thick arrows indicate osteoblasts, and thin arrows indicate osteocyte in bone lacuna.

By using X-ray, we found that both groups experienced significantly enhanced ossification on the 30th day compared with those on the 15th day (P<0.05). Besides, significantly more bones formed in the study group than those in the control group (P<0.05). Relative area ratios of new bones are summarized in Table 2 and Figure 4, and the fillings of new fibrous tissues after toluidine blue staining are shown in Figure 5. After 30 d, 60 d and 90 d, subcutaneous implants were removed from the rabbits and analyzed for new bone formation. The toluidine blue staining showed that each group displayed increased bone formation over the 90-day time period. High neovascularization was observed in the pores of β-TCP in each group. A maximal and robust bone formation was presented in the study group. Osteoblastic cells were lining the surface of newly formed bone. In some areas, considerable numbers of osteocytes in the newly formed thickened bone on the β-TCP were seen in the study group.

**Table 2:** Relative area ratios of new bones (%).

	n	15 d^1^	30 d^2^	45 d^3^	60 d^4^
Study A	12	2.95±1.03	10.27±2.68	47.71±7.29	79.65±9.88
Control B	12	1.00±0.57	7.29±1.80	16.27±3.19	48.17±7.73
t		−5.92	−6.28	−10.27	−16.88
P		0.0378	0.0189	0.0077	0.0001

P_1_, P_2_, P_3_ and P_4_<0.05, suggesting the differences are statistically significant which increased on the 30th day.

### Real-time quantitative PCR and RT-PCR results

**Figure 6:**
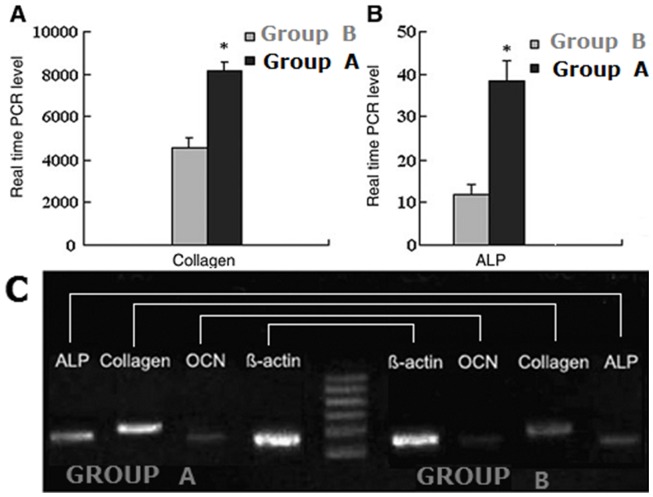
Real-time quantitative PCR and RT-PCR results. Type I collagen (A) and ALP (B) levels of the BMSCs in the two groups detected on the 14th day using real-time quantitative PCR (n = 6, mean ± SD), *P<0.05; (C) type I collagen, ALP and OCN mRNA levels of the BMSCs in both groups detected on the 14th day using RT-PCR.

The Real-time quantitative PCR and RT-PCR results are presented in Figure 6. The results showed that VEGF 165 and could increase significantly ALP activity/protein, OCN content, and mineral formation of BMSCs. indicated that VEGF 165 could provide a better environment for osteogenic differentiation of BMSCs. Actually, the structure and composition of β-TCP had an effect on the osteogenic differentiation of BMSCs, ALP, and type I collagen, and OCN are important osteoblastic markers and are expressed at different maturation stages of the osteoblasts. Real-time quantitative PCR and RT-PCR results further proved that BMSCs seeded onto β-TCP with VEGF 165 had significantly higher expression of ALP, type I collagen, and OCN mRNAs. It suggested that VEGF 165 had effected sufficiently osteogenic differentiation of BMSCs seeded on β-TCP.

### Vascularization results

**Figure 7:**
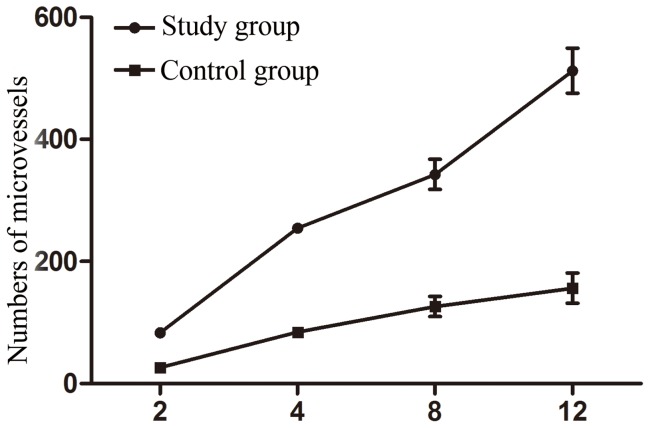
Numbers of microvessels at each time point.

**Figure 8:**
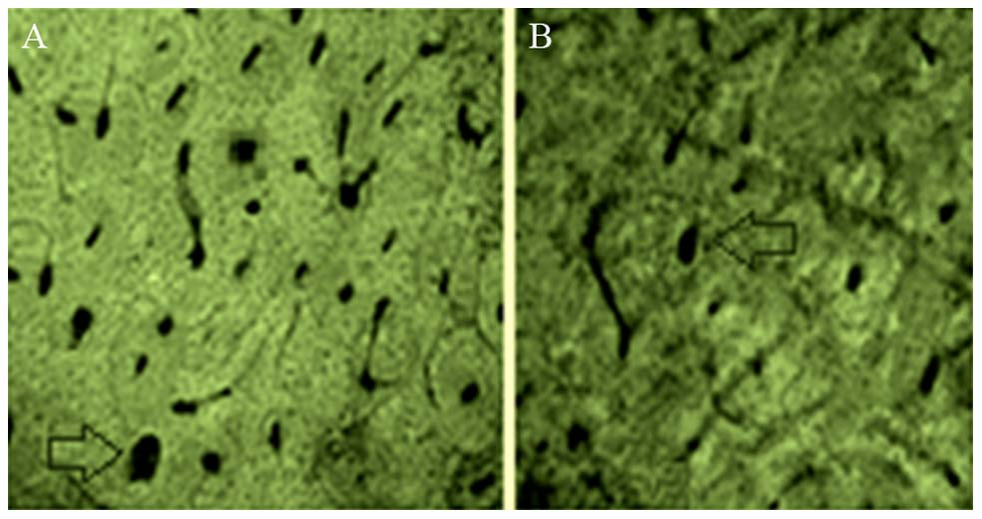
Cross sections of undecalcified ground bones 60 days after vascular casting. Blank arrows indicate cross sections of the blood vessels filled with casting agents. (A) Study group; (B) control group.

**Figure 9:**
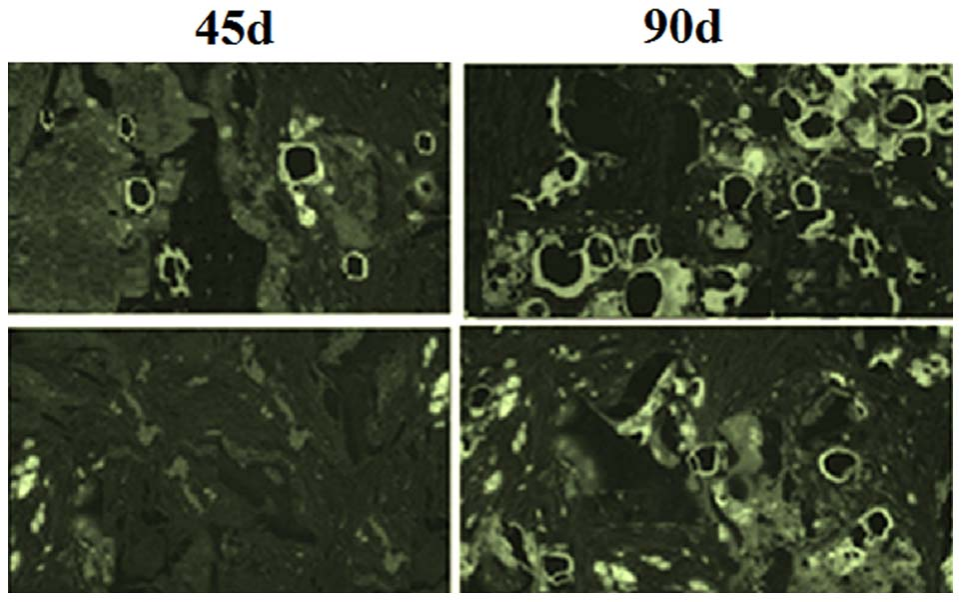
CD31 immunohistochemical staining results on the 45th and 90th days. Upper two: study group; lower two: control group.

There were more microvessels with elapsed time (P<0.05), and the number of microvessels in the study group (34.75±8.23) was significantly higher than that of the control group (19.22±2.76) (P<0.05). Numbers of microvessels at each time point are listed in Table 3 and Figure 7, and the vascularization results of the two groups are exhibited in Figure 8. CD31 staining (Figure 9) shows that the number of new blood vessels in the study group was significantly higher than that of the control group.

**Table 3:** Numbers of microvessels at each time point.

	n	15 d^1^	30 d^2^	45 d^3^	60 d^4^
Study A	12	5.76±1.03	20.23±3.79	34.75±8.23	50.89±9.84
Control B	12	1.90±0.67	10.27±2.27	19.22±2.76	30.80±7.64
T		−7.92	−10.43	−16.95	−19.88
P		0.0382	0.0096	0.001	0.001

P_1_, P_2_, P_3_ and P_4_<0.05, suggesting the differences are statistically significant which increased on the 45th day.

Microvessels were clearly visible, and blood vessels reticulated along Haversian and Volkmann's canals. Meanwhile, nutrient vessels grew into endostea and periostea. There were Haversian canals in the middle as well as in internal and external lamellae, with blood vessels reticulating along Haversian and Volkmann's canals.

## Discussion

We herein verified that tissue engineering bones were prone to forming and repairing after being transfected with human VEGF gene. In other words, ossification was dramatically accelerated because BMSCs were supplied with abundant oxygen and bloods. Thirty days after vascular casting, both groups underwent significantly elevated VEGF expressions, as well as enhanced ossification and vascularization. Moreover, the study group constantly had better results than the control group did (P<0.05). In this study, VEGF managed to accelerate ossification by boosting vascularization.

Hanahan [18] reported that angiogenesis-promoting and angiogenesis-inhibiting factors mutually regulated the growth of blood vessels delicately under physiological conditions. However, disequilibrium undesirably led to pathological angiogenesis. The factors promoting angiogenesis include VEGF, bFGF (basic fibroblast growth factor), ANG (angiogenin), PDGF (platelet-derived growth factor), TGF-β (transforming growth factor-β) and EGF (epidermal growth factor), etc., and those inhibiting angiogenesis include angiostatin, endostatin and platelet factor-4, etc. [19], which are dimeric oligoglycoproteins linked by follicular astrocyte-produced disulfide bonds. VEGF, which is closely associated with vascularization and matrix mineralization during bone distraction, is capable of accelerating bone formation by enhancing the proliferation and differentiation of vascular endothelial cells and preosteoblasts. VEGF can specifically function upon EC as a highly conservative dimeric glycoprotein derived from disulfide-bonded subunits. As an essential promoter in the midst of angiogenesis, VEGF first binds corresponding receptors by regulating heparin-like molecules, thus inducing self-phosphorylation and selectively activating mitogen-activated protein kinase. Therefore, EC was stimulated to reproduce through mitosis. In addition, raising the permeability of blood vessels and particularly microvessels provides nutrients for cell growth and establishment of a network of new capillaries [20]. In case of low oxygen concentration and action of oncogene products, cells produce hypoxia-inducible factor 1 that evidently facilitates expression of VEGF gene and biological activity of VEGF as a transcription factor [21].

Biologically speaking [22], VEGF is a heparin-binding dimeric glycoprotein that is able to induce the proliferation of endothelial cells, to promote the differentiation, migration, amplification and accumulation of osteocytes, and to augment the activity of alkaline phosphatase, being consistent with the results in this study. VEGF was highly expressed on the 30th day (approximately the 4th week) when considerable microvessels formed, i.e. vascularization was facilitated from the 15th to the 30th day, thus rendering observation on from the 20th to the 28th day reasonable when ossification was promoted simultaneously. In the meantime, healing was well maintained because vascularization and ossification synchronized.

Furthermore, we applied genetic engineering, a prospective protocol, in this study by transfecting VEGF 165 gene with adenovirus. To make significant progress in molecular biology [23], we used adenovirus for transfection because VEGF had short half-life (5 min) during intravenous infusion [24]. Transfection thus continuously induced vascularization and ossification before healing by maintaining high VEGF expression.

Given BMSCs are vulnerable to death owing to insufficient oxygen supply, many scholars have been devoted to circumventing the issues by vascular bundle grafting, transfection of VEGF, and grafting of VEGF in combination with osteocytes [25]–[27]. It has previously been reported that [28], [29] VEGF was highly expressed on the 30 day and decreased thereafter when blood vessels began to atrophy. Besides, the results of study subjects did not differ from those of controls. In this study, differences between the two groups remained significant after approximately 9 weeks. However, this study was not continued until the 24th week due to time limit, which motivates us to conduct further studies.

In summary, compared with those untransfected, VEGF-transfected engineering bones helped healing of New Zealand white rabbits more effectively by promoting vascularization and ossification, thus being worthy of clinical application in future.
